# Unraveling the molecular dynamics of wound healing: integrating spatially resolved lipidomics and temporally resolved proteomics

**DOI:** 10.1007/s00216-025-05865-5

**Published:** 2025-04-24

**Authors:** Hongxia Bai, Alejandra Suarez Arnedo, Yining Liu, Tatiana Segura, David Muddiman

**Affiliations:** 1 Biological Imaging Laboratory for Disease and Exposure Research (BILDER), Department of Chemistry, North Carolina State University, Raleigh, NC 27695 USA; 2 Department of Biomedical Engineering, Duke University, Durham, NC 27708 USA

**Keywords:** Wound healing, Mass spectrometry imaging, 3D, IR-MALDESI, Multi-omics

## Abstract

Understanding the spatial–temporal molecular dynamics of wound healing is crucial for devising effective treatments. Three-dimensional mass spectrometry imaging (3D MSI) enables the comprehensive visualization of molecular distribution throughout skin layers, offering valuable insights into the wound healing process. However, traditional 3D MSI often faces challenges in maintaining data integrity and accurate image registration in the third dimension. To address this, we employed infrared matrix-assisted laser desorption electrospray ionization (IR-MALDESI), a hybrid ambient ionization technique capable of sequential imaging through consecutive ablation events for precise 3D image reconstruction. Herein, 3D IR-MALDESI MSI was used to compare the lipidome of fresh-frozen wound samples at three stages of wound healing (inflammation, proliferation, and remodeling) with the healthy skin of SKH- 1 mice. Supplementing this data with a refined LC–MS-based proteomics protocol on selected wound biopsies, our integrated approach deepens our understanding of the molecular intricacies inherent in tissue regeneration.

**Graphical Abstract:**

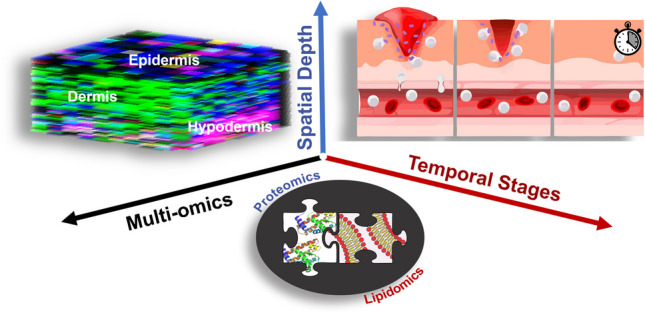

**Supplementary Information:**

The online version contains supplementary material available at 10.1007/s00216-025-05865-5.

## Introduction

As the body’s most extensive organ, the skin provides a critical barrier against external threats such as pathogens and various stimuli. Its integrity, once disrupted by injury, must be promptly restored to preserve its protective function. The complex process of wound healing requires the concerted interplay of diverse cell types, including neutrophils and macrophages, and unfolds in three overlapping stages: inflammation, proliferation, and remodeling [1]. A disruption in this progression may culminate in chronic wounds, ulcers, or excessive scarring [2].

Chronic wounds afflict millions of people, particularly with a rising incidence of diabetes and aging population in the USA; subsequently, this propagates to a significant economic burden, exceeding $25 billion annually for treatment measure [3]. While recent advancements have shed light on certain aspects of wound healing, a comprehensive molecular understanding remains a challenge. Despite numerous research efforts to understand the overall patterns of proteins involved in wound healing [4–6], a comprehensive understanding of the interaction between proteins and other compounds such as lipids remains incomplete.

Mass spectrometry, particularly mass spectrometry imaging (MSI), is revolutionizing the approach to analytical challenges [7]. While MSI adeptly elucidates the spatial distribution of a variety of molecules in a single label-free experiment [8], it has a significant drawback: sampling bias from studying 3D biological events with 2D sections. The newer three-dimensional mass spectrometry imaging (3D MSI) is emerging as a method to capture more comprehensive sample data [9]. Traditional 3D mass spectrometry imaging (MSI) approaches often rely on serial sectioning to generate volumetric data, a process that can result in significant loss of biological information and challenges in achieving accurate 3D image reconstruction [10]. Alternatively, techniques leveraging continuous ablation beams, such as ion or laser beams, have been developed. These methods enable direct sampling of the sample’s surface while simultaneously exposing fresh layers for subsequent imaging, thereby bypassing the need for physical sectioning. Secondary ion mass spectrometry (SIMS) has been a pioneering approach in this domain, utilizing dynamic ion beams to facilitate high-resolution molecular depth profiling and 3D imaging of biological systems [9, 11, 12]. However, SIMS is often constrained by its small molecular ion generation and challenges in handling complex biological matrices. In contrast, infrared matrix-assisted laser desorption electrospray ionization (IR-MALDESI) facilitates 3D imaging with laser ablation, generation of a 3D molecular profile by continuously imaging the same region while ablating through the sample depth [13, 14]. Preliminary studies have validated IR-MALDESI’s suitability for biological studies, demonstrating its efficacy on both over-the-counter pharmaceuticals (hard) and tissue samples (soft) without the need for tedious sample preparation [13, 14].

Understanding wound healing is intricate and requires a holistic multi-omics approach for comprehensive molecular assessment. Lipids, despite their quintessential role in skin function, have their spatial dynamics in wound healing underrepresented in the literature. Phospholipids, particularly glycerophospholipids and sphingolipids, have recently been reported as bioactive lipids essential for intra- and intercellular communication and maintaining cell membrane structure [15]. Eicosanoids, especially those from the cyclooxygenase (COX) pathway, are crucial in this context. They influence various stages of wound healing, from platelet aggregation to tissue remodeling, with molecules like thromboxane A2 (TxA2), prostaglandin E2 (PGE2), and prostaglandin D2 (PGD2) playing pivotal roles [16]. The significance of arachidonic acid and its metabolites (e.g., leukotrienes and prostaglandins) in maintaining balance and managing inflammation is undeniable [17]. Furthermore, recent research highlights the emergence of pro-resolving mediators like resolvins in response to wound stimuli, underscoring the evolving understanding of lipid-mediated wound healing [18]. Simultaneously, the use of proteomic techniques, drawing on tissue biopsies and wound exudates, has significantly enriched our knowledge of wound healing [19, 20]. These methods reveal potential therapeutic targets and markers for healing progression with a recurring focus on proteins like elastase, growth factors, fibronectin, and matrix metalloproteases such as MMP− 2 and MMP- 9 [19, 21].

Despite these independent endeavors into lipidomics and proteomics, integrative studies that combine these data remain sparse. Recent advancements in multi-omics approaches have demonstrated their ability to bridge this gap, offering a more comprehensive understanding of molecular dynamics. Studies integrating spatially resolved metabolomic and lipidomic data with proteomics have revealed cell-type-specific metabolic and lipidomic variations, highlighting tissue heterogeneity during physiological and pathological processes [22–24]. For instance, 3D mass spectrometry imaging has illuminated the interplay between metabolic and lipid pathways in host–pathogen interactions [25], while large-scale efforts like the Human BioMolecular Atlas Program (HuBMAP) have underscored the importance of spatially resolved omics in mapping cellular diversity and molecular interactions [26]. These findings underscore the potential of integrated approaches in studying complex biological phenomena, including wound healing.

In this study, we investigated the process of wound healing using a full-excisional murine healing model with SKH- 1 mice. We employed an integrative approach, combining 3D IR-MALDESI MSI for lipidomic analysis and bottom-up proteomics by LC–MS, to examine the different stages of wound healing. Through 3D MSI, we investigated lipid distribution, and proteomics allowed us to identify proteins potentially involved in wound healing. While this work offers preliminary molecular insights into the healing process, future studies with expanded datasets and additional biological replicates will be critical to fully validate these findings and translate them into therapeutic strategies for managing wounds.

## Experimental details

### Materials

In this study, an 11-week male SKH1 mouse model was purchased from Charles River Laboratories. Biopsy punch pens of various sizes used to create the wounds, were purchased from Integra Miltex. The following materials were sourced from Thermo Fisher Scientific (Wilmington, DE) and used for proteomic analysis: 1 M Tris–HCl solutions (pH 7.5 and 8.0), sodium chloride (NaCl), ammonium bicarbonate (ABC), Pierce™ trypsin protease (MS grade), LC/MS grade water (H_2_O), LC/MS grade acetonitrile (ACN), LC/MS grade formic acid (FA), Vivacon 500 30 kD MWCO filters, and the Pierce™ BCA Protein Assay Kit. Other reagents, such as urea, dithiothreitol (DTT), and iodoacetamide (IAA) were procured from Bio-Rad (Hercules, CA).

### Wound healing study

Male SKH- 1 Elite mice (8–12 weeks old) were housed in a centralized animal facility at Duke University. We used an excisional splinted wound protocol as described in previous publications [27, 28]. The mice were anesthetized with 4% isoflurane (1.5–2% isoflurane for maintenance), injected with Buprenorphine SR (1 mg/mL, 0.5 μg per g of mouse weight) subcutaneously before surgery, and placed on a warming pad. Four clean, well-defined wounds were created along the middle of the animal’s back using sterile 5 mm biopsy punches after the dorsal surface was sterilized with iodine and ethanol. PDMS ring splints with a 7-mm wide window were adhered around the wounds to prevent contraction and allow for healing through re-epithelialization and granulation. Wounds were treated with clinically used standard treatment Woun’Dres [28]. Tegaderm dressings were applied to the back to prevent the wounds from drying out. The animals were weighed and checked every other day and housed individually in cages. All procedures were approved by the Duke University Institutional Animal Care and Use Committee and followed the NIH Guide for the Care and Use of Laboratory Animals.

### Experimental design and sample collection

Following wounding, the mice were kept for 4, 14, and 21 days before being euthanized using Carbon dioxide (CO_2_) inhalation and cervical dislocation. 7-mm biopsy punch samples of the wounded areas with adjacent tissue and samples of unwounded healthy skin were collected and immediately frozen in a bath with acetone and isopentane on foil-taped glass slides for lipidomic analysis. Additionally, 1.5 mm biopsy punch samples were collected for proteomic analysis using low-binding 1.5 mL tubes. All samples were stored in a − 80 °C freezer until the mass spectrometry imaging measurements were conducted.

**Fig. 1:**
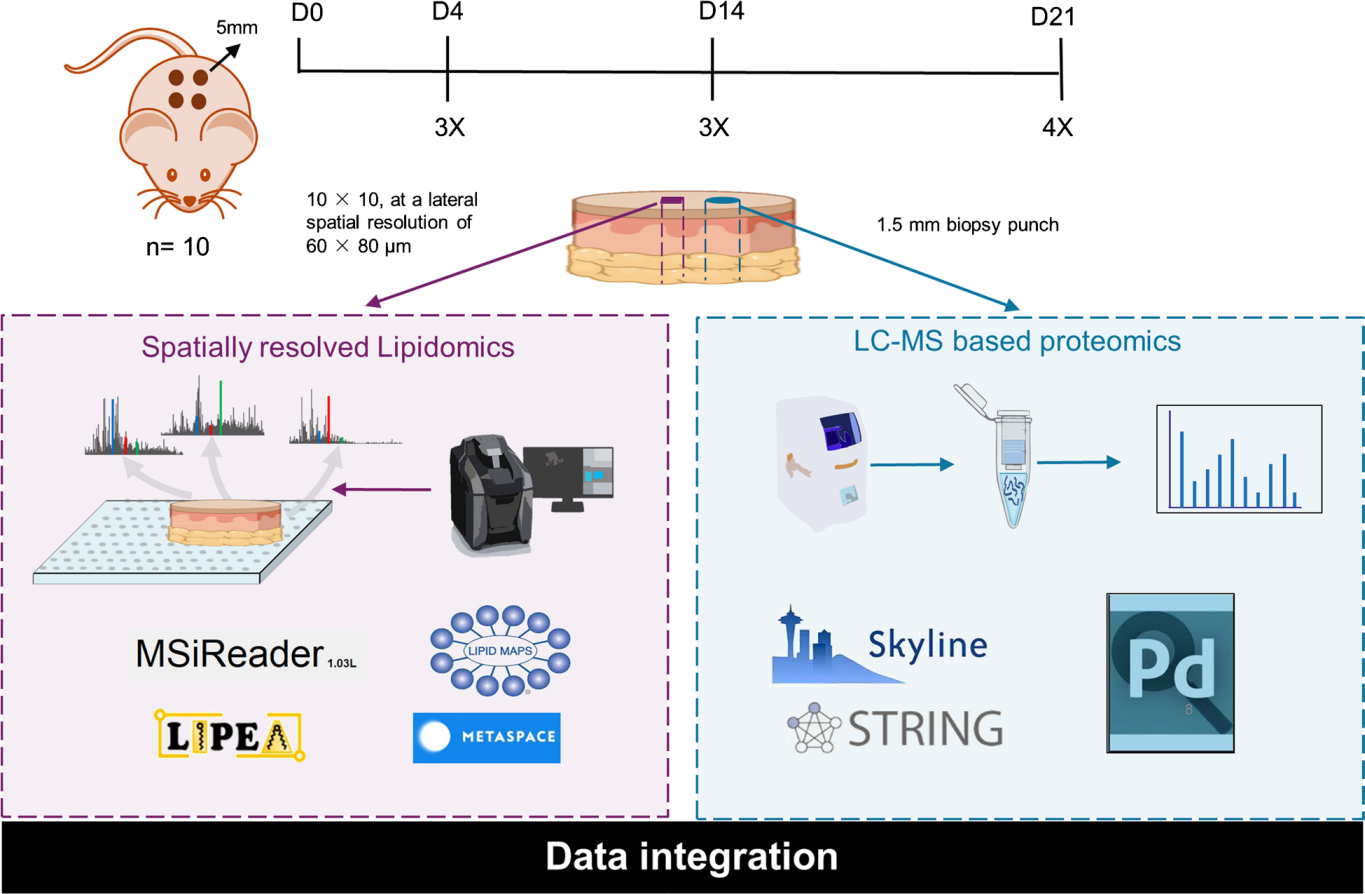
Overall workflow for wound healing experiments: Dermal excisional wounds were created using a 5-mm biopsy punch and harvested at specific time points (4, 14, and 21 days) to represent inflammation, proliferation, and regeneration stages. The number of replicates collected at each time point are specified. From each harvested sample, a 1.5 mm biopsy was taken from the wound center and processed using a typical proteomics workflow involving homogenization, extraction, and FASP. The remaining wound sample underwent analysis using IR-MALDESI MSI to investigate the spatially resolved lipidome. Integration of the proteomics and lipidomic data provided insights into the wound healing process

The detailed experimental workflow is illustrated in Fig. 1. Dermal excisional wound samples were obtained at three critical time periods: day 4, representing the inflammation phase; day 14 for the proliferation phase; and day 21, indicative of the regeneration phase [28]. Three samples were collected for day 4 and 14, four samples for day 21 and five samples of unwounded healthy skin. A subsequent biopsy of 1.5 mm was extracted from the epicenter of each wound. This biopsy underwent comprehensive proteomics sample processing, including tissue homogenization, protein extraction, and the filter-aided sample preparation (FASP) method, to obtain purified peptide samples for bottom-up proteomics analysis. The residual portion of each wound sample was subjected to an analysis via IR-MALDESI MSI, a technique specifically chosen for its ability to map and define the lipidome with spatial accuracy.

### IR-MALDESI imaging platform

An in-house developed IR-MALDESI source was integrated with an Orbitrap Exploris 240 mass spectrometer (Thermo Fisher Scientific, Bremen, Germany) for imaging purposes [29]. The ion source employed water/ice matrix-assisted laser desorption, complemented by post-ionization through electrospray. An infrared laser (JGM Associates, Inc., Burlington, MA, USA) with an output energy between 0.4 and 0.5 mJ and a wavelength of 2.97 µm was used to ablate the sample surface. The ablated material was then directed into an orthogonal electrospray plume, which was generated with a flow rate of a 1.5 µL/min and a voltage of 3500 V. Mass spectra acquisition was performed in positive mode, ranging from *m/z* 150–600. The system was operated with the EASY-IC enabled, automatic gain control (AGC) deactivated, and a mass resolving power set at 240,000_FWHM_ at *m/z* 200. For optimal synchronization with ion generation, an injection time of 25 ms, and a handshake delay time of 140 ms were utilized.

The imaging process was controlled by the custom software RastirZ [30]. Imaging was completed across 40 layers with a region of interest (ROI) of 10 × 10, at a lateral spatial resolution of 60 × 80 µm. To prevent the laser from losing focus during continuous ablation, manual stage adjustments were executed to ensure the laser remained within its depth of focus. After ablation, the sample craters’ depth was gauged using a Keyence confocal microscope (VK-X1100, Keyence, Itasca, IL, USA). This measurement facilitated determining the necessary *z*-resolution adjustment to uphold the laser’s focus.

### LC–MS based proteomics workflow

The LC–MS-based proteomics workflow began with the homogenization of a 1.5-mm biopsy, derived from the same sample as MSI, using a GenoGrinder (Spex Sample Prep, NJ, USA). Homogenization parameters included a force of 1400 g, alternating between 30-s grinding cycles and 30-s rest intervals, repeated for a total of four cycles. For optical protein solubility, three 2.9 mm diameter beads were used combined with 100 µL of 50 mM ABC and 1% SDC. Following homogenization, protein concentrations were measured with a BCA assay, and an aliquot equivalent to 15 µg of total protein was subject to a modified filter-aided sample preparation workflow [31, 32].

In brief, samples underwent reduction in 50 mM dithiothreitol with 0.1 M Tris–HCl (pH 8.0) at 56 °C for 30 min. Afterward, samples were placed onto a 30 k Da MWCO filter and centrifuged to remove surplus dithiothreitol. Next, alkylation was performed by treating the samples with 64 µL of 200 mM iodoacetamide in 8 M urea in 0.1 M Tris–HCl pH 8.0 for 1 h at room temperature in the dark. Excess iodoacetamide was eliminated via centrifugation, and retained proteins were thrice washed using 2 M urea, 10 mM calcium chloride in 0.1 M Tris–HCl (pH 8.0). This was followed by pH adjustment and purification using 0.1 M Tris–HCl (pH 7.5). The samples then underwent overnight digestion at 37 °C using 0.5 µg of trypsin, resulting in tryptic peptides, which were stored at − 20 °C pending LC–MS/MS analysis.

The proteomics measurements were performed using a Thermo EASY nano LC 1200 interfaced with an Orbitrap Exploris 480 mass spectrometer (Thermo Fisher Scientific, Bremen, Germany). The setup employed a “trap-and-elute” configuration with specific column dimensions: a 0.075 mm × 20 mm C_18_ trap column and a 0.075 mm × 250 mm C_18_ analytical column. The analysis involved injecting 2 µL of the sample peptides (1 µg/µL in 2% acetonitrile in water with 0.1% formic acid) and maintaining a nanoLC flow rate of 300 nL/min. Elution of peptides was achieved using a 135-min gradient with varying concentrations of Mobile Phase B (MPB). The eluted peptides were ionized in the ion source with 1.9 kV applied, and data acquisition was performed using full MS scans and data-dependent acquisition (DDA) MS/MS. The other instrument settings included a scan range of 375 to 1600, a resolving power of 120,000_FWHM_ (at *m/z* 200), a 120 ms maximum injection time, an HCD normalized collision energy set at 30%, and dynamic exclusion intervals lasting 20 s periods.

### Data analysis

For MSI, the XCalibur raw data were converted to imzML format using an open-source *raw to imzML converter* software [33]. Once converted, the imzML files were subsequently analyzed using the MSiReader software (v1.03) for both data visualization and statistical analysis [34, 35]. The “Peakfinder” algorithm in this software was employed to identify unique ions based on two constraints. Firstly, a peak had to be present in more than 80% of the interrogated zone and present in less than 20% of the reference zone. Secondly, if a peak was present in more than 20% of the reference zone, the average abundance ratio (reference: interrogated) must exceed 2. This approach allowed for the identification and characterization of specific ions of interest in the MSI datasets.

Potential lipids were annotated at the formula level using LipidMaps [36], with a set mass accuracy constraint of ± 2.5 ppm. After this, the identified potential lipids underwent lipids pathway enrichment analysis using the LIPEA tool (https://lipea.biotec.tu-dresden.de/home). The primary aim of this analysis was the identification of significantly perturbed pathways in relation to the lipids detected, utilizing the Kyoto Encyclopedia of Genes and Genomes (KEGG) database. The Over-Representation (or enrichment) Analysis (ORA) was employed to determine pathways that were overrepresented compared to the background *Mus musculus* (*M. musculus*) lipids database. This comprehensive analysis provided insights into the functional pathways associated with the identified lipids in the study.

In the proteomics analysis, the raw data was searched against the *M. musculus* fasta database using Proteome Discoverer 2.5 (Thermo Scientific, San Jose, CA) with the SEQUEST HT algorithm. The search parameters included trypsin (full) as the digesting enzyme, allowing for a maximum of 3 missed trypsin cleavage sites, a precursor mass tolerance of 5 ppm, and a fragment mass tolerance of 0.02 Da. Peptide validation was performed using Percolator, with a *q*-value threshold set to achieve a minimal false discovery rate (FDR) of less than 0.01. Proteome Discoverer was also utilized to analyze differentially expressed proteins among different sample groups, generating label-free relative quantification data. Both unique and razor peptides were used for relative quantification, without any normalization or scaling applied. To test the hypothesis of fold change based on the summed protein abundance, an analysis of variance (ANOVA) test was performed. Classification, functional enrichment, and protein interaction analysis of the proteins were performed using STRING (v12). Expression analyses like Volcano plots, and Venn’s diagram, were made using VolcanoNoseR [37], and InteractiVenn [38] respectively. Additional graphs were plotted using GraphPad Prism software 9 (GraphPad Software, San Diego, CA, USA).

## Results and discussion

Wound healing represents a multifaceted physiological response, marked by a dynamic interplay of cellular activities and molecular pathways [39]. To navigate this complexity, the present approach harnessed the capabilities of IR-MALDESI for lipidomic profiling and LC–MS/MS for proteomic analysis. Utilizing IR-MALDESI, the 3D resolved lipidomic configuration of mouse skin samples had been previously mapped, offering a detailed insights into the volumetric distributions of varied lipids [14]. Building on this established methodology, we applied IR-MALDESI to wounded skin specimens to explore lipidomic dynamics across different stages of wound healing. This approach offers a preliminary perspective on lipid behavior during the healing process and lays the foundation for further integrative studies.

### Depth resolution and system repeatability in IR-MALDESI imaging

Establishing an accurate *z*-resolution is pivotal for maintaining a consistent focus during 3D imaging with IR-MALDESI. This is particularly vital given the Gaussian profile of the laser, which tends to lose focus over time, especially as material is progressively ablated from the samples. To quantify the *z*-resolution, laser ablation was carried out on healthy skin biopsies for 5, 10, and 20 layers. The subsequent ablated craters were analyzed with the VK-X1100 Keyence Confocal microscope. Graphing the ablation depth against the respective layer numbers, the derived slope gave an average depth per layer, effectively denoting the *z*-resolution. Utilizing a laser energy between 0.4 and 0.5 mJ, the *z*-resolution was found to be approximately 9 µm (Supplemental Fig. 1). This crucial information now serves as a guide for the *z*-stage movement in 3D imaging, preserving laser focus integrity throughout the process.

**Fig. 2:**
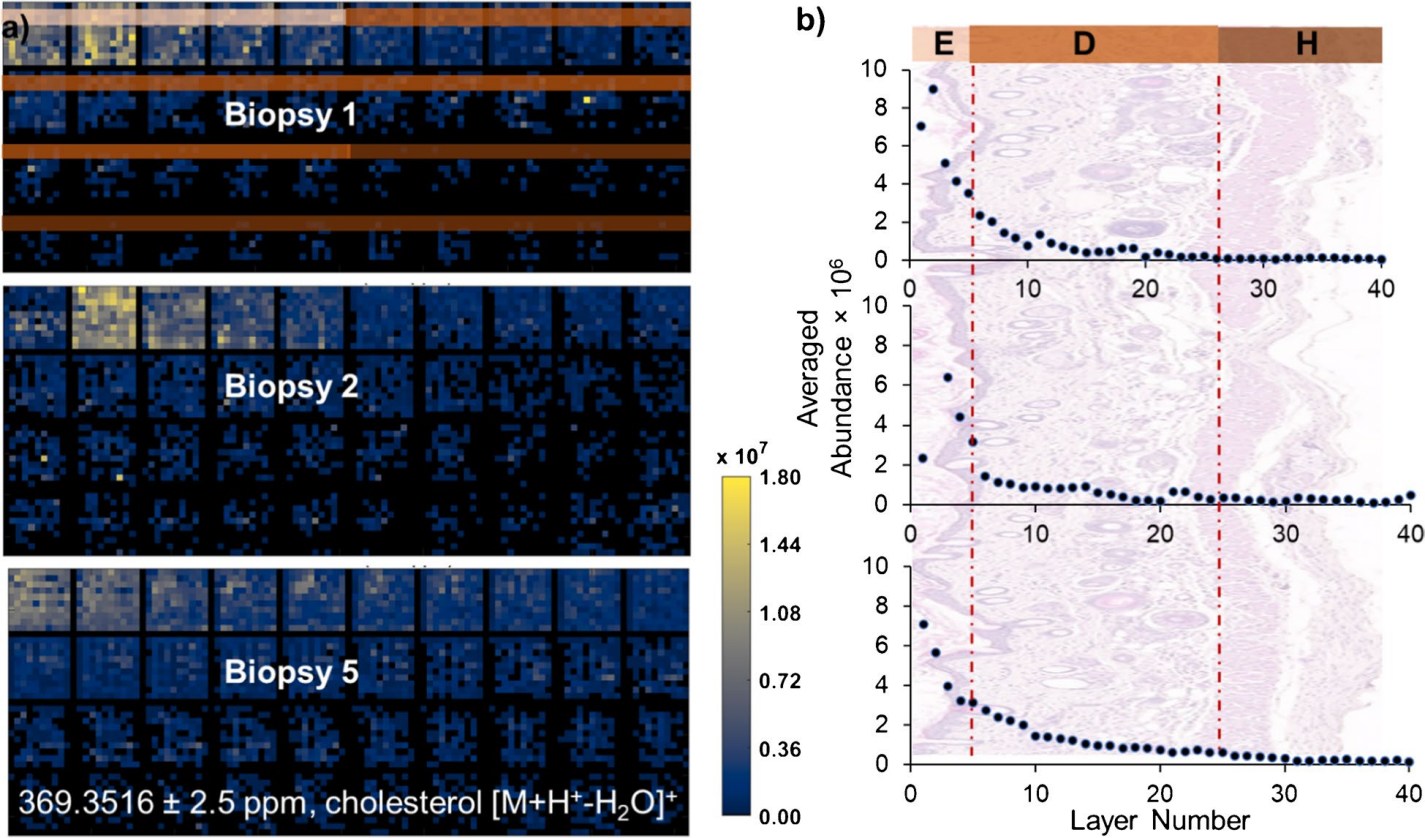
MSI analysis of cholesterol with IR-MALDESI, shown as [M + H^+^-H_2_O]^+^ at *m/z* 369.3516. **a** Heatmaps display the 3D distributions of cholesterol across three biopsy samples, demonstrating reproducibility despite inherent biological variability. **b** Graphical representation of cholesterol abundance against layer number. The labels E, D, and H correspond to the epidermis, dermis, and hypodermis layers, respectively, and correlate to the appropriate skin anatomy in the background of the graph

Having established the *z*-resolution, a detailed 40-layer imaging experiment was performed on biopsies from three distinct healthy mice to validate the repeatability of the IR-MALDESI imaging methodology. Cholesterol ion, identified as [M + H + -H_2_O]^+^ with *m/z* 369.3516, served as a reference molecule for this validation. Heatmaps, as depicted in Fig. 2a, illustrate the cholesterol abundance and its consistent distribution across different skin layers. Despite originating from diverse samples, the cholesterol distribution patterns exhibit remarkable consistency, showcasing a descending abundance gradient from the epidermis through to the dermis and finally the hypodermis. However, owing to the inherent unevenness of murine skin, minor discrepancies in cholesterol levels were noted within the initial three layers across the samples, as visualized in Fig. 2b. These minor variations notwithstanding, the overarching abundance trends were consistent, underscoring the repeatability of the IR-MALDESI imaging system for biological applications. It is worth noting that in the biopsy from the first mouse, periodic abundance disruptions were observed at every 10-layer interval, potentially stemming from variations in laser fluence due to intermittent stage adjustments.

### Lipidomic profile during wound healing

**Fig. 3:**
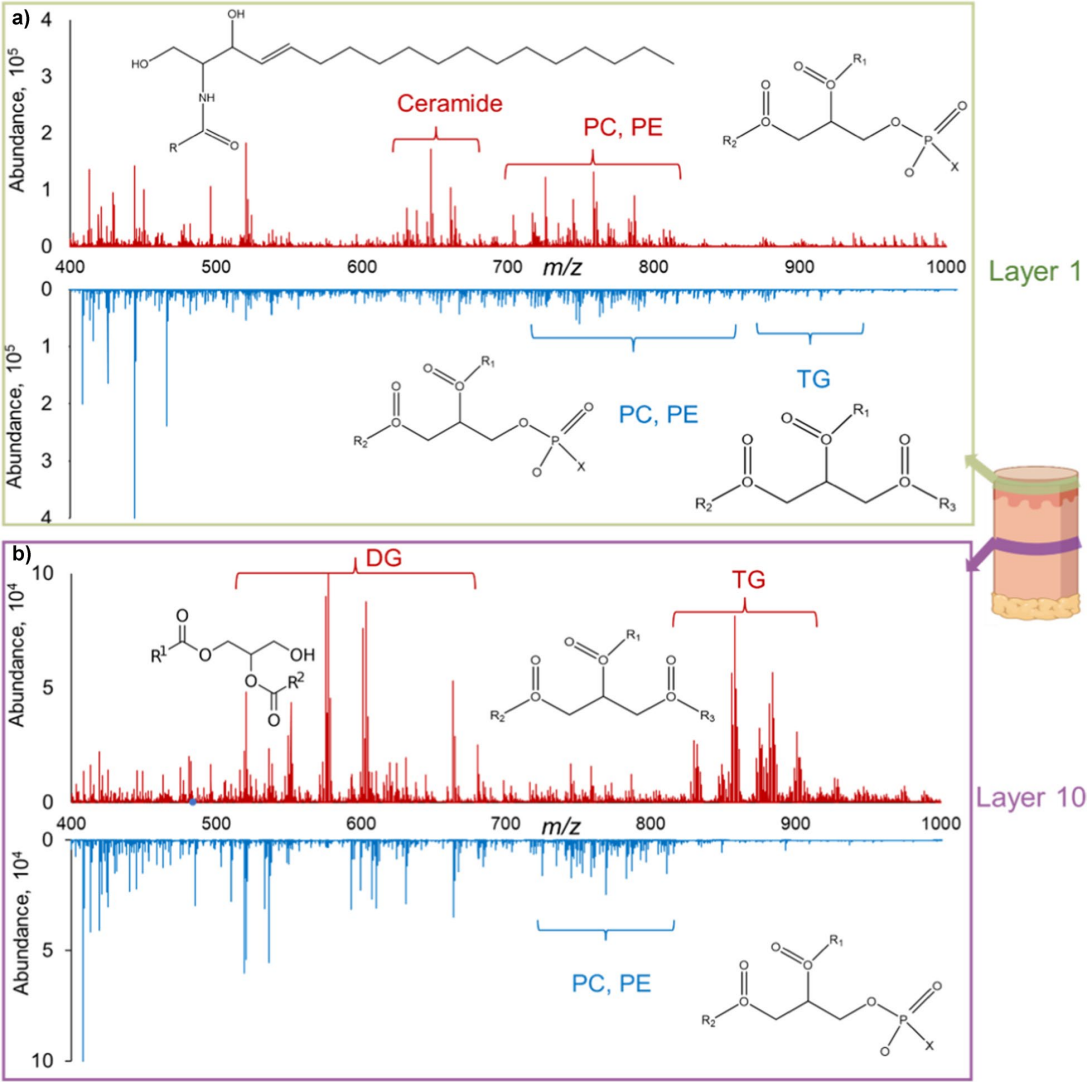
Comparison of mass spectra obtained from a 40-layer 3D MSI experiment. The epidermis in layer 1 (**a**) versus the dermis in layer 10 (**b**). Distinct lipid profiles are evident when comparing wounded samples (red) with the healthy control (blue). Specifically, in the epidermis of layer 1, wounded samples show heightened levels of ceramide, phosphatidylcholine (PC), and phosphatidylethanolamine (PE) relative to the healthy biopsy. In contrast, the dermis of layer 10 reveals increased abundance of diglycerides (DG) and triglycerides (TG) in the wounded skin, but diminished levels of PE and its species. These variances in lipid composition underscore the potential modifications in lipid metabolism and signaling intrinsic to the wound healing process

Initial analysis centered on the comparison of spectra between wounded controls and their healthy counterparts during inflammation. Distinct discrepancies were noted in the abundance profiles of ceramide (Cer), phosphatidylcholine (PC), and phosphatidylethanolamine (PE) within the epidermal region, as depicted in Fig. 3a. Ceramides, which constitute approximately half of the stratum corneum’s mass when combined with cholesterol and free fatty acids, are pivotal to the structural and functional integrity of this outermost epidermal layer. A compromised stratum corneum, due to ceramide dysregulation, can manifest as disrupted skin barrier functions, imbalanced homeostasis, and escalated trans-epithelial water loss. Beyond their structural role, ceramides also modulate inflammatory responses, being integral membrane components that react to exogenous stimuli [40]. The heightened ceramide abundance in wounded skin may suggest an amplification of these processes. The heatmap for a representative ceramide, Cer (42:2), showcases its pronounced presence in wounded sites relative to healthy controls (Supplemental Fig. 2a). This particular ceramide species, previously identified in macrophages of mice, is reportedly accentuated during macrophage activation [41]. While ceramide and its derivatives have been integral in various cellular activities, ceramide- 1-phosphate (C1P) stands out for its roles in fibroblast mobility, mast cell activation, and inflammation mediation [42]. In our study, a subtle increase in C1P was observed in the wound environment compared to the normal tissue (Supplemental Fig. 2b). These observations align with prior research suggesting that the dynamics of C1P during wound healing might differentiate wound tissue from normal tissue. However, the differences in our dataset were not as pronounced as might be expected based on previous findings, underscoring the need for further investigation.

In the dermal layer, a pronounced abundance of sebaceous glands is observable, consistent with the elevated levels of diglycerides (DG) and triglycerides (TG) depicted in Fig. 3b. DGs, as key intermediates in lipid metabolism, are involved in the synthesis of TGs and may also influence cellular signaling pathways that regulate inflammation and tissue repair during wound healing. However, the specific mechanisms by which DGs contribute to this process remain unclear and warrant further investigation. TGs are known for their roles in proliferation and adaptive immunity, but still remains underexplored concerning their specific contribution during wound healing. Notably, TGs can be converted into acetyl-CoA, providing ATP to meet the energy demands of the wound repair process. Past efforts have even documented the utility of lipid concoctions rich in TG in facilitating wound repair [43].

To gain deeper insights into the lipidomic alterations in wounded samples, unique ions from representative layers were delineated and subjected to pathway analysis, revealing overarching shifts across the skin’s various layers. Lipid candidates were identified using LipidsMaps based on molecular formula matches (Supplemental Table 1) and were evaluated through functional enrichment analysis, with the dominant pathways delineated in Table 1. Significant pathway enrichment was predominantly observed in the epidermis, including glycerophospholipid (GPL) metabolism, choline metabolism, ether lipid metabolism, fat digestion and absorption, and sphingolipid (SP) signaling. Given the established roles of GPLs in both intra- and intercellular communications, their prominence in wounded samples aligns with expectations. Intriguingly, analysis ofthe deeper layers of dermis and hypodermis reveals a relative surge in unique ion levels but a paucity in enriched pathways. This observation suggests that, while lipidomic changes occur throughout the wound environment, the epidermis serves as the primary metabolic hub during wound healing, likely reflecting its active role in barrier restoration and cellular regeneration.

**Table 1:**
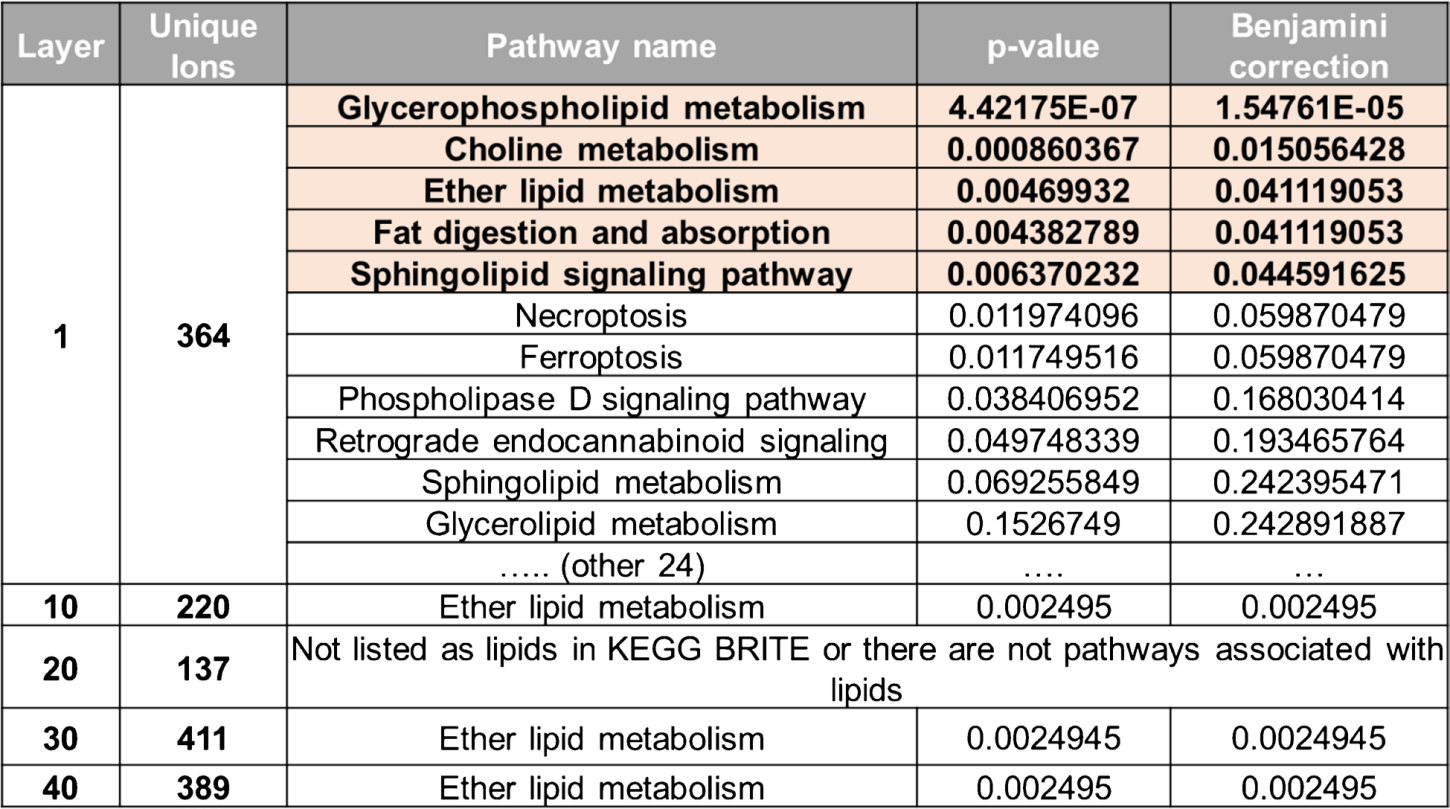
Pathways enriched in distinct skin layers identified from lipidomic analysis. This table summarizes the key pathways identified from lipidomic analysis of wounded samples. Observed pathway enrichment was predominantly found in the epidermis, suggesting its role as a primary metabolic hub during the wound healing process. These findings represent preliminary insights and require further validation to confirm their biological significance

### Proteomics in wound healing

While lipidomics offers a detailed view of lipid distribution and composition across skin layers, proteomics provides insight into the intricate proteomics molecular responses of the skin during different phases of wound healing. Proteomics has been an essential tool in wound healing research, shedding light on complex molecular pathways and mechanisms that guide the healing process [44, 45]. Through evaluating the roles of inflammatory mediators, growth factors, extracellular matrix proteins, and immune cell activity, previous proteomic studies have advanced our understanding of wound progression and highlighted potential therapeutic targets [45].

In this study, our proteomic analysis aimed to elucidate the temporal variations in protein expression throughout wound healing process under standard treatment with Woun’Dres [28]. A secondary objective of our study was to explore the potential molecular mechanisms underlying those changes. By identifying differential protein expression, we sought to characterize how the wound healing process evolves across its distinct stages (Supplemental Table 2).

**Fig. 4:**
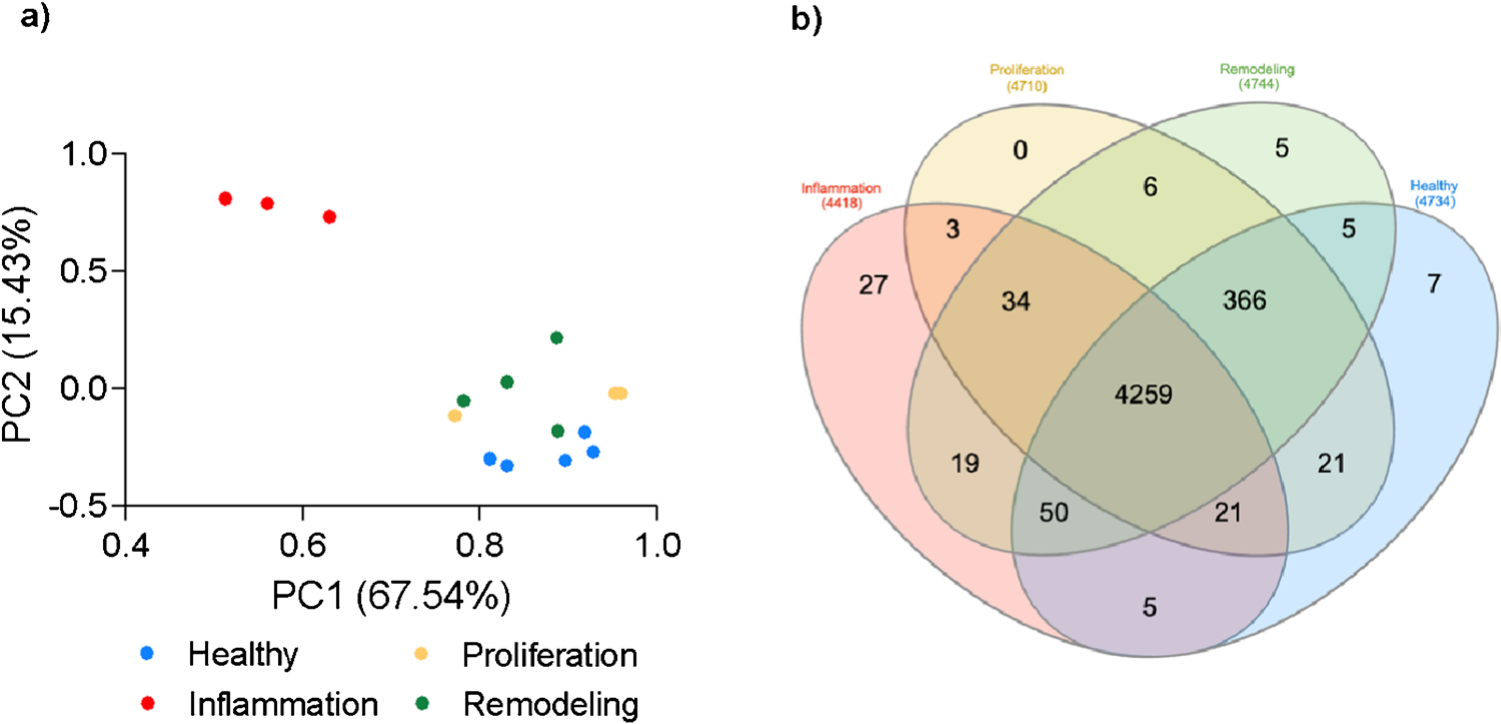
Distinct proteomic landscapes across wound healing stages visualized through PCA and Venn’s diagram. **a** This PCA plot captures the unique proteomic signatures characteristic of each wound healing stage. Samples are clustered based on their molecular interactions and expression profiles. This figure underscores the inflammation phase as a pivotal moment in wound healing, driving metabolic surges and orchestrating subsequent stages of tissue repair and regeneration. **b** Venn’s diagram comparing each stage of wound healing process with healthy unwounded skin

Principal component analysis (PCA) revealed clear proteomic distinctions among the different stages of wound healing (Fig. 4a**)**. Each stage, defined by specific molecular and cellular interactions, formed distinct separate clusters in the PCA plot, echoing findings from previous studies that analyzed wound healing stages in human samples [46]. Notably, the inflammation phase exhibited a unique proteomic signature, characterized by differential expression of pro-inflammatory mediators, immune cell markers, and enzymes involved in oxidative stress. This phase, marked by a surge in metabolic activity, is a critical juncture in wound healing. It sets the stage for the following processes, coordinating the interplay of proteins and molecular pathways to initiate immune response, tissue repair, and regeneration [46, 47].

Although clear clustering was observed with PCA, Venn diagram analysis (Fig. 4b) highlights that out of the 4828 proteins identified in this study, 4259 (88.2%) are expressed across all stages of wound healing and healthy skin. This finding underscores that it is the relative abundance and dynamic changes within the protein network, rather than the mere presence of individual proteins, drive the temporal phase of wound healing [46].

Based on Fig. 4b and Supplemental Table 3, we have identified 27 uniquely expressed proteins during the inflammation stage. Among these, several members of the Small Proline-Rich Protein (SPRR) family—Sprr2 h, Sprr2k, Sprr2b, Sprr2 g, and Sprr2 d—were identified. These proteins are known to play crucial roles in maintaining epidermal homeostasis and providing protection against bacterial invasion, underscoring their relevance in skin barrier function [48].

Additionally, three members of the Rho GTPase Activating Protein family—Arhgap32, Arhgap33, and Arhgap31—were uniquely expressed during the inflammatory phase. Rho GTPase Activating Proteins are known to regulate Rho GTPases, which are involved in cytoskeletal remodeling during cell wound repair [49]. Additionally, Rho GTPases have been implicated in the migration, activation, differentiation, and apoptosis of immune cells in other inflammatory diseases contexts, such as inflammatory bowel disease [50].

Interestingly, two proteins that are uniquely expressed in inflammation are associated with lipid metabolism: Pla2 g4e (Phospholipase A2, Group IVE) and Apolipoprotein M (ApoM). ApoM is an apolipoprotein that regulates sphingosine- 1-phosphate (S1P), which plays a critical role in cell signaling related to inflammation and wound healing. Pla2 g4e, a phospholipase, is responsible for the release of arachidonic acid, a precursor for inflammatory mediators [51]. These findings suggest potential roles for lipid metabolism in coordinating inflammatory responses during wound healing, though further studies are needed to fully elucidate these mechanisms.

**Fig. 5:**
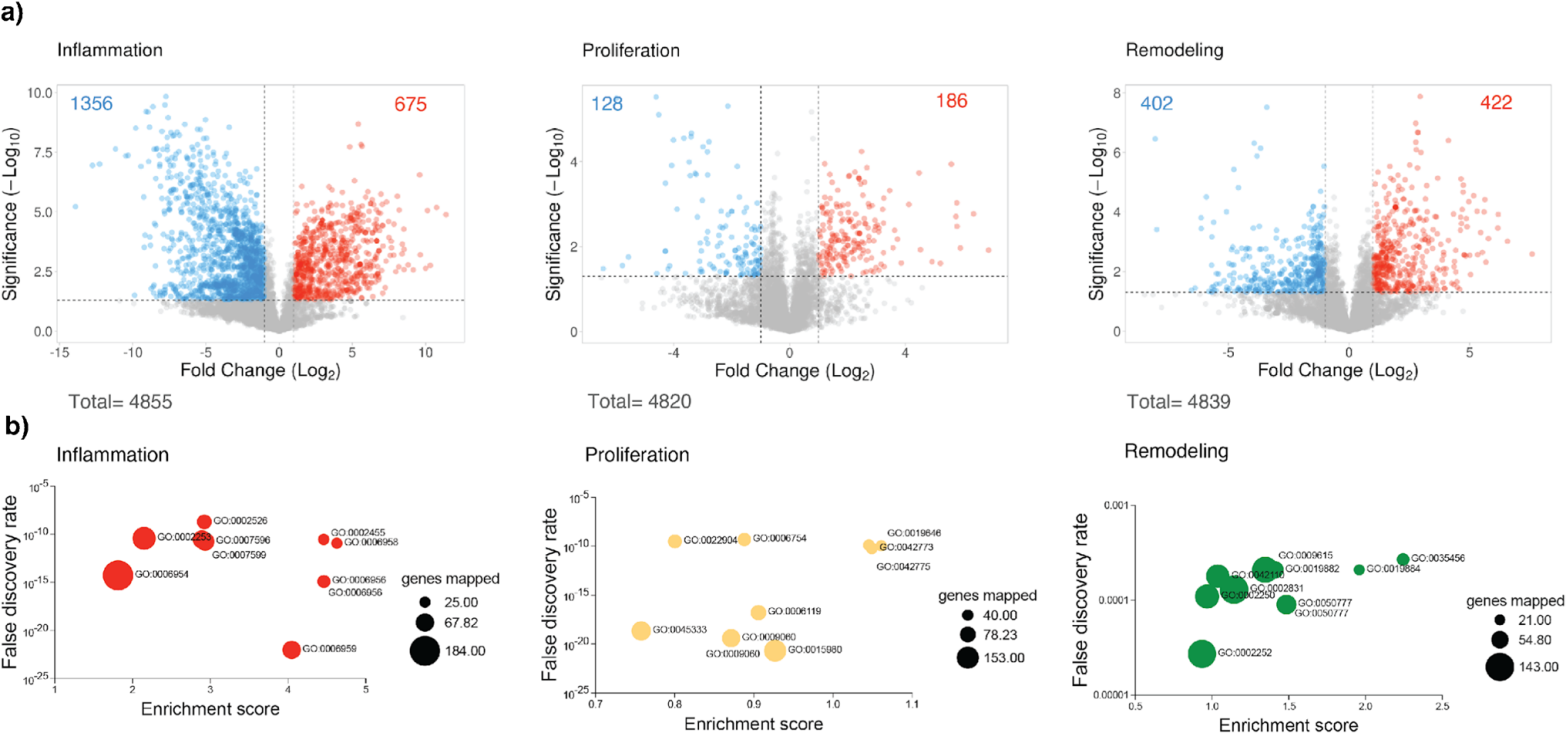
Differential protein expression across wound healing stages. **a** Volcano plots showing differential protein expression across the phases of wound healing. Wounded samples compared to normal tissue during the inflammation, proliferation, and regeneration phases of wound healing. Each dot represents a protein, with its position indicating the extent of change (x-axis) and the statistical significance of that change (y-axis). The plots provide insight into the unique molecular responses and potential therapeutic effects of treatments in the context of wound healing progression. **b** Bubble plots displaying the biological functions (Gene ontology) with the highest upregulation in each stage of wound healing

We employed volcano plots to compare treated samples with healthy controls using a significance threshold of p-value < 0.05 and a fold change of 2 (Fig. 5a and Supplemental Table 4). The inflammation phase exhibited the most notable changes during healing, with 1,356 downregulated and 675 upregulated proteins. In comparison, the proliferation stage showed 128 downregulated and 186 upregulated proteins, while the remodeling stage exhibited 402 downregulated and 422 upregulated proteins.

Gene ontology analysis using Ranked-Based Testing (Fig. 5b and Supplemental Table 5) highlighted stage-specific enrichment patterns. During the inflammation stage, 248 GO-terms were significantly enriched. These terms were primarily associated with immune response (GO:0002253, GO:0006954), bias towards a humoral response (GO:0006959, GO:0002455), as well as pathways associated with the coagulation cascade (GO:0007596) and hemostasis (GO:0007599). These results align with the cellular biology process described in this stage, where hemostasis creates a blood clot as the primary barrier following injury, and an immediate immune response triggers an acute humoral and inflammatory activity [39].

The proliferation stage exhibited 106 significantly enriched GO-terms, with the most upregulated terms associated with cellular respiration (GO:0045333) and ATP biosynthesis process (GO:0006754). These results are consistent with active metabolic activity during this stage, driven by the migration and recruitment of fibroblasts, endothelial cells, and keratinocytes to the wound site [52].

Finally, the remodeling phase displayed 148 significantly enriched GO-terms, with the most upregulated terms linked to adaptive immune response (GO:0002250) and T-cell activation (GO:0042110). This stage reflects a shift in cytokine profiles and the presence of adaptive immune cells, which help promote tissue regeneration and regulate the behavior of cells such as fibroblasts. These process contribute to the deposition and the remodeling of extracellular matrix components during wound repair [53].

### Integration of proteomics and lipidomics

The integration of lipidomics and proteomics provides a lens through which we can offers a valuable framework for exploring the molecular dynamics of wound healing, particularly the interplay between lipids and proteins during the healing process. Proteomic analysis highlighted the significance of proteins involved in inflammation, prompting a focused examination of the upregulated protein set during this phase. Harnessing the LipidsMaps Proteome Database (LMPD) enabled us to identify lipid-related proteins among this upregulated set, revealing key actors in lipid metabolism.

Beyond their well-recognized roles as cellular membrane constituents, lipids play pivotal roles in signaling, energy storage, and regulation of cellular processes [54]. Our integrated lipids-related proteins analysis highlights the multifaceted functions of these lipid functions. Specifically, we identified proteins associated with the biosynthesis of phosphatidylcholine (PC) and sphingolipids (SP), both of which are critical components of cellular membranes. In additional to biosynthesis, lipid transport proteins, such as fatty acid-binding proteins (FABPs) and lipid transfer proteins, underscore the intricate mechanisms facilitating efficient lipid transport within cellular environments.

Moreover, our dataset reveals the significance of lipid-mediated signaling, particularly through the proteins involved in the phosphoinositide 3-kinase (PI3 K) pathway. The pronounced presence of these proteins during the inflammation aligns with the activities of neutrophils and macrophages, key immune cells that play central roles in the wound healing process. While traditionally associated with host defense and infection, these immune cells are also deeply interconnected with lipid-mediated signaling processes, further emphasizing their diverse functional roles during inflammation and tissue repair.

**Fig. 6:**
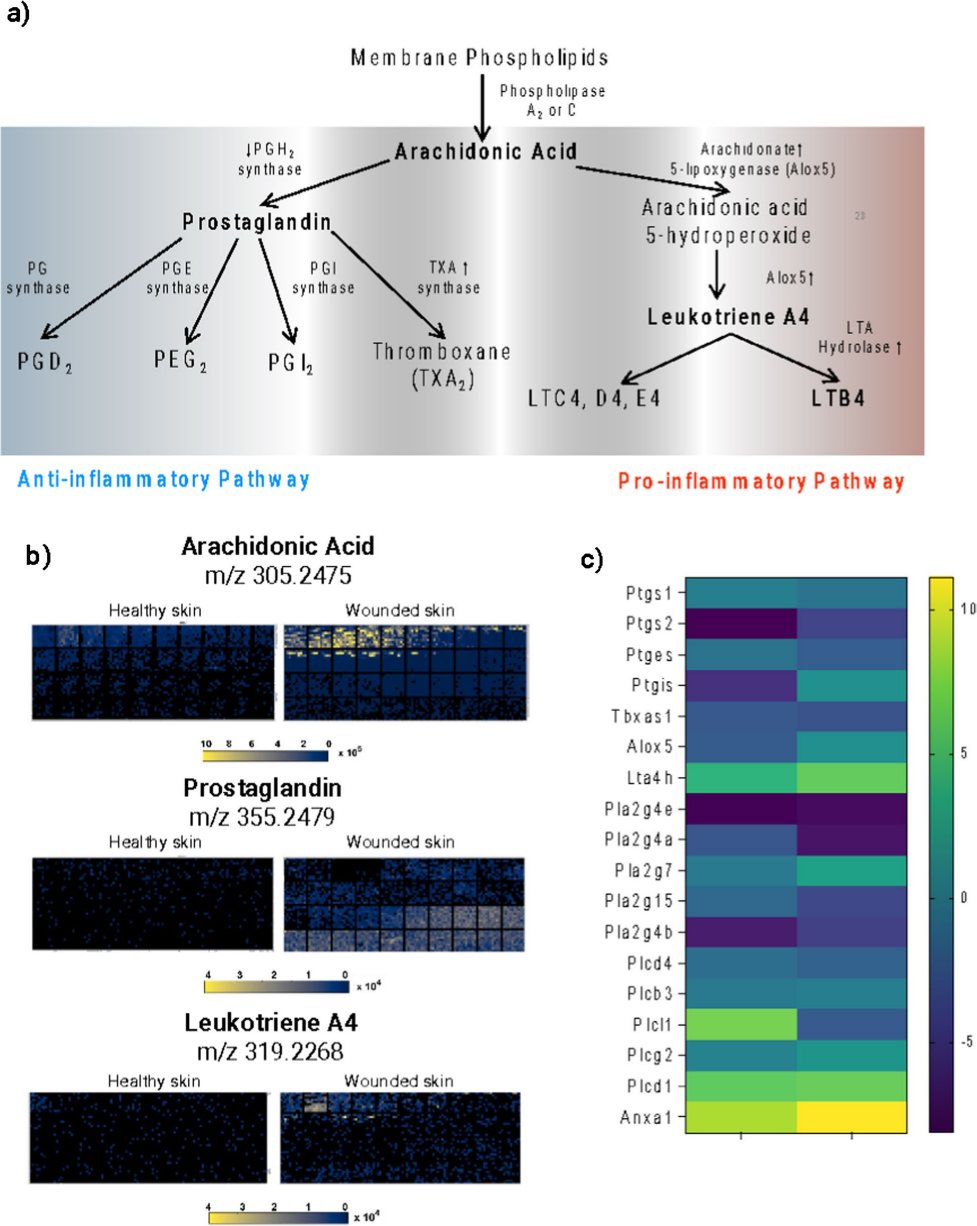
Significance of the arachidonic acid pathway in wound healing. **a** schematic representation of the arachidonic acid pathway, highlighting its bifurcation into two distinct sub-pathways involved in inflammation regulation. **b** 3D heatmaps present the spatial distribution of differentially regulated lipids within the arachidonic pathway. These heatmaps provide a comparative analysis, emphasizing the differences in microenvironments observed in healthy against wounded skin. **c** Heatmap of the enzymes associated with arachidonic acid pathway

In this analysis, a notable discovery was a group of proteins marked italicized in Table 2, which all belong to the eicosanoid pathway. As depicted in Fig. 6a, this pathway is centered around the transformation of arachidonic acid, a polyunsaturated fatty acid liberated from cell membranes via the activity of phospholipase A2 or C. This pathway has been previously implicated in wound healing process by numerous studies [55, 56]. Arachidonic acid serves as a precursor for various lipid mediators. Collaborating with PGH2 synthase, it can be converted into substances such as prostaglandins, thromboxanes, prostacyclins, and various other prostanoids, which majorly undertake anti-inflammatory functions [55]. On another route, the enzyme arachidonate 5-lipoxygenase directs arachidonic acid to form an intermediate, arachidonic acid 5-hydroperoxide (5-HETE), which subsequently turns to leukotriene A4 and B4 (LTB4) [56]. These pro-inflammatory lipid mediators contribute to the initiation and propagation of inflammation by activating platelet aggregation. The elevated abundance of enzymes involved in both anti-inflammatory and pro-inflammatory pathways emphasizes the complex interplay and signaling network within the wound healing process.

**Table 2:** Upregulated lipid-related proteins during the inflammation stage in wound control. The majority are lipoproteins, with those presented in italics representing enzymes integral to the arachidonic acid pathway

Protein name	Function
Aldehyde dehydrogenase 9, subfamily A1	Enables oxidoreductase activity, acting on the aldehyde or oxo group of donors, NAD or NADP as acceptor
*Arachidonate 5-lipoxygenase activating protein*	*Synthesis of leukotrienes*
1-acylglycerol- 3-phosphate O-acyltransferase 4	Enables 1-acylglycerol- 3-phosphate O-acyltransferase activity
Annexin A1	Phospholipase A2 inhibitor activity
Apolipoprotein A-IV	Anti-inflammatory
Apolipoprotein B	Lipids Transporter
Apolipoprotein B receptor	Enables very-low-density lipoprotein particle receptor activity
Apolipoprotein C-I	Activation of esterified lecithin cholesterol
Apolipoprotein C-IV	Lipids Transporter
Apolipoprotein E	Lipids Transporter
Apolipoprotein H	Regulatory role in coagulation, lipids binding
α− 2-glycoprotein 1, zinc	Stimulates lipolysis
Biotinidase	Recycle of vitamin biotin
Clusterin	Lipid transport, modulate the activity of leptin
Coenzyme Q2 homolog, prenyltransferase (yeast)	Glycerol metabolic process and ubiquinone biosynthetic process
Cellular retinoic acid binding protein I	Retinoic acid-mediated differentiation and proliferation processes
DnaJ (Hsp40) homolog, subfamily A, member 1	Stimulates ATP hydrolysis
GC vitamin D binding protein	Actin binding activity and calcidiol binding activity
Group specific component	Transports vitamin D and its metabolites
Glycosylphosphatidylinositol specific phospholipase D1	Releasing the attached protein from the plasma membrane
Glutathione peroxidase 3	Response to lipid hydroperoxide
Lipopolysaccharide binding protein	Elicit immune responses
*Leukotriene A4 hydrolase*	*Arachidonic acid metabolism*
Macrophage scavenger receptor 1	Enables amyloid-beta binding activity
Neutrophil cytosolic factor 1	Active in phagocytes
Phospholipase A2, group VII (platelet-activating factor acetylhydrolase, plasma)	Phospholipids binding activity
Phospholipid transfer protein	Enables lipid transfer activity
Protein kinase C, δ	Regulate immunity (regulates the production of interleukin- 12 (IL- 12) p40/70 in both macrophages and dendritic cells)
Retinol binding partner 4	Transferring retinol
Paraoxonase 1	Enables arylesterase activity
*Prostaglandin I2 (prostacyclin) synthase*	*Synthesis of cholesterol, steroids and other lipids*
Serine (or cysteine) peptidase inhibitor, clade A, member 6	Major transport protein for glucorticoids and progestins
STT3, subunit of the oligosaccharyltransferase complex, homolog A (S. cerevisiae)	Enable dolichyl-diphosphooligosaccharide-protein glycotransferase activity
Solute carrier family	Membrane transport

The lipid profiles shown in Fig. 6b, along with additional data, illustrate significant changes occurring in the samples. Notably, arachidonic acids exhibit heightened levels across all wounded specimens, indicating a shift in lipid metabolism associated with the wound healing process. The wounded samples also show increased levels of inflammation-promoting leukotriene A4, suggesting a more pronounced inflammatory response. This observation is consistent with the expected physiological reaction to wounding. The differential expression observed in the heatmaps of lipids from healthy and wounded skin can be explained by the differences in enzyme expression presented in Fig. 6c. Specifically, the increased expression of Alox5 and LTA4 h enzymes correlates with the pro-inflammatory environment in wounded samples, as evidenced by the higher levels of leukotriene A4. Alox5 (5-lipoxygenase) plays a crucial role in the biosynthesis of leukotrienes from arachidonic acid [57], while LTA4 h (Leukotriene A4 hydrolase) catalyzes the conversion of leukotriene A4 to leukotriene B4, a potent inflammatory mediator [58]. The upregulation of these enzymes explains the elevated leukotriene A4 levels observed in the wounded samples. These findings underscore the complex and tightly regulated inflammatory response during wound healing, highlighting how molecular-level changes in lipid metabolism and enzyme activity contribute to the broader physiological response to wound healing.

The evolution of multi-omics approaches is at the forefront of reshaping how we understand complex physiological processes like wound healing. The ability to overlay different data landscapes offers a holistic perspective, enabling a detailed exploration of the intricate relationships between cellular events and molecular signaling pathways. Our research, by focusing on the integration of spatial resolved lipidomics and temporal proteomics, presents a methodology with potential applicability to a myriad of biological phenomena, not limited to wound healing alone.

A key insight from our study underscores the pivotal role of inflammation in wound healing, as illuminated by our proteomics analysis. The findings emphasize the necessity of regulating inflammation for optimal recovery. Additionally, the spatially resolved lipidomics study sheds light on the epidermis as an intensely active participant in the wound healing process. Utilizing the entire wound sample for lipidomics could have overlooked these specific epidermal contributions, highlighting the indispensability of 3D analytical methodologies in discerning spatial heterogeneities.

It is important to note that the arachidonic acid pathway highlighted has been previously implicated in the inflammatory stage of wound healing by numerous studies [55, 56]. Our findings do not represent a new discovery of this pathway’s involvement, but rather provide additional validation through a multi-omics approach. By combining spatially resolved lipidomics with temporally resolved proteomics, we offer a more comprehensive view of how this established pathway functions within the broader context of wound healing. This integrated approach allows us to observe the interplay between lipid mediators and their associated proteins, providing a deeper understanding of the molecular dynamics at play.

However, it is pertinent to note that while our emphasis on lipid-related proteomics has revealed valuable insights, it only scratches the surface of the potential that integrated multi-omics holds. One potential limitation of our study is the chosen approach itself. Vertical analyte delocalization remains a potential concern in 3D MSI experiments. Future studies should incorporate reverse-orientation imaging to validate the spatial distribution of analytes from the hypodermis to the epidermis. Although not performed here, such approaches would provide a more robust assessment of analyte localization and mitigate potential artifacts. Also, by prioritizing lipid-related proteomics, we may have inadvertently sidelined other important proteins or pathways that could be equally instrumental in the wound healing process. Furthermore, other multi-omics layers—like transcriptomics, genomics, or metabolomics—could also provide invaluable context and depth, creating a more comprehensive narrative. This work, while methodologically focused, would benefit immensely from being underpinned by more extensive biological data. Another limitation is the sample size. Greater biological replicates would lend more statistical power and robustness to our findings. While our results are promising, broadening the sample pool in future research will better cement the relationships and trends we have identified. Our approach demonstrates the potential of integrated multi-omics not just for pathway discovery, but for providing deeper insights into complex biological processes. By combining spatial lipidomics with temporal proteomics, we offer a methodology that could be applied to various biological phenomena during wound healing.

## Conclusions

The study of wound healing was conducted using a comprehensive multi-dimensional approach, encompassing spatial, temporal, and multi-omics analyses. The spatially resolved lipidomic profiles provided insights into the distinct molecular compositions of the epidermis, dermis, and hypodermis during the wound healing process. Enrichment analysis further emphasized the significant biological activity observed in the epidermis, highlighting its crucial role in wound healing. Temporally resolved proteomics revealed dynamic changes across all three treatment groups, with a clear indication that the inflammation stage represents the most active phase in the wound healing process. The integration of multi-omics data was exemplified through a robust examination of arachidonic acid and its metabolic pathway, showcasing the potential of this approach for comprehensive molecular investigations. Moving forward, future efforts will be directed towards enhancing the system and advancing data interpretation techniques, enabling a deeper understanding of wound healing mechanisms.

## Supplementary Information

Below is the link to the electronic supplementary material.

Supplementary Figure 1& 2 (DOCX 846 KB)

Supplementary Table 1 (XLSX 52 KB)

Supplementary Table 2 (XLSX 3332 KB)

Supplementary Table 3 (XLSX 72 KB)

Supplementary Table 4 (XLSX 1017 KB)

Supplementary Table 5 (XLSX 18 KB)
