# Supplementary material for: Unraveling the molecular dynamics of wound healing: integrating spatially resolved lipidomics and temporally resolved proteomics
Source: Anal Bioanal Chem. 2025 Apr 24;417(15):3299–314. doi: 10.1007/s00216-025-05865-5 (PMC12122605; doi:10.1007/s00216-025-05865-5)
Supplement: Supplementary file 1 — Supplementary Figure 1& 2 (DOCX 846 KB) [file 216_2025_5865_MOESM1_ESM.docx]

**Unraveling the Molecular Dynamics of Wound Healing: Integrating**

**Spatially Resolved Lipidomics and Temporally Resolved Proteomics**

Hongxia Bai^a^, Alejandra Suarez Arnedo^b^, Yining Liu^b^, Tatiana Segura^b^, David Muddiman^a^

*^a^Biomolecular Imaging Laboratory for Disease and Exposure Research (BILDER), Department of Chemistry, North Carolina State University, Raleigh, NC 27695 USA; ^b^Department of Biomedical Engineering, Duke University, Durham, NC 27708 USA*

**Submitted to:** *Analytical and Bioanalytical Chemistry*

**SUPPLEMENTARY MATERIAL**

**Keywords:** Wound healing, Mass spectrometry imaging, 3D, IR-MALDESI, Multi-omics

***Author for Correspondence**

David Muddiman, Ph.D.

Biomolecular Imaging Laboratory for Disease and Exposure Research

Department of Chemistry

North Carolina State University

Phone: 919-513-0084

Email: [dcmuddim@ncsu.edu](mailto:dcmuddim@ncsu.edu)

***
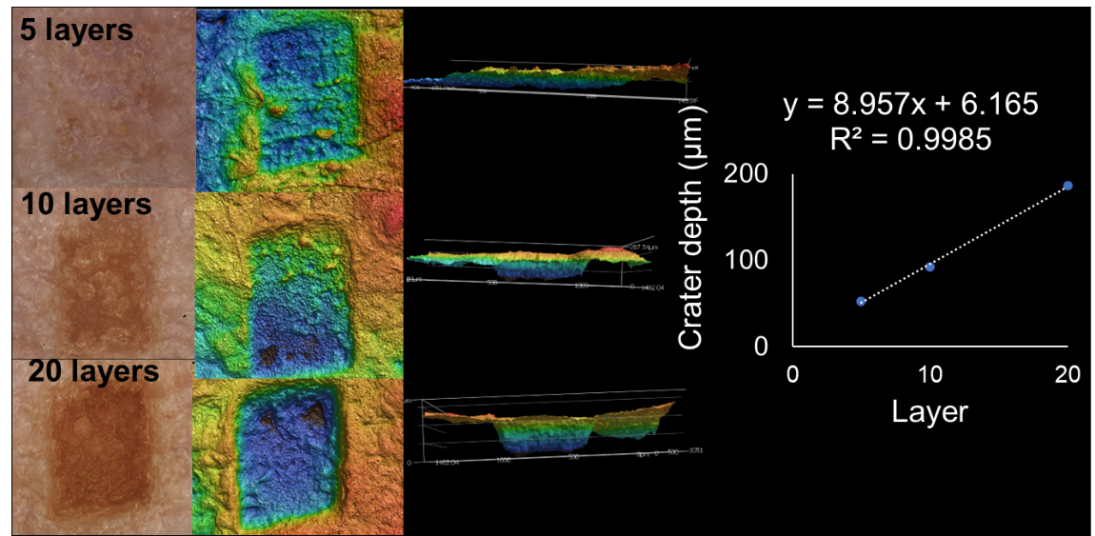
***

**Supplemental Figure 1.** Depth resolution determination via continuous ablation. The resolution is gauged by the depth to which the laser penetrates and, specifically, the depth of laser ablation for each layer. A depth profiling of ablated craters in nude mouse skin was executed by ablating the tissue in increments of 5, 10, and 20 layers. Subsequent characterization was carried out using a Keyence confocal microscope. With a burst laser energy ranging between 0.4-0.5 mJ, the depth resolution for the profiles was estimated at approximately 9 µm.


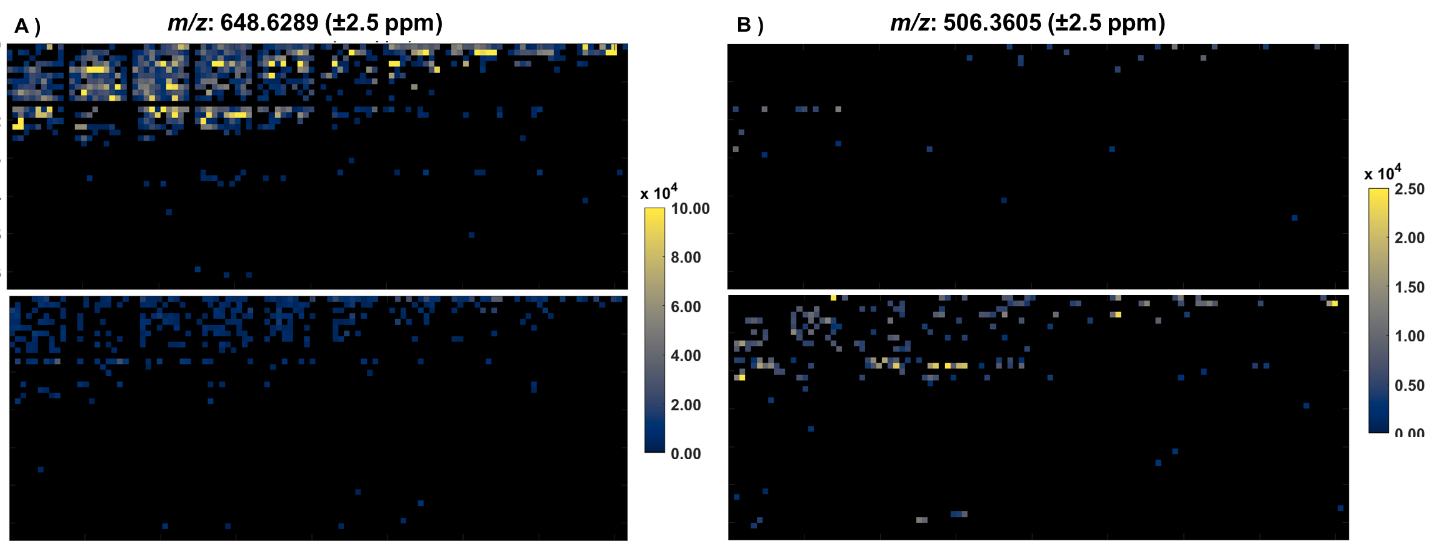


**Supplemental Figure 2.** Supplemental Figure 2. Differential lipid abundance in normal vs. wound tissue during the inflammation stage. Heatmaps depict the relative levels of a) ceramide (([C_42_H_81_NO_3_+H^+^]^+^, m/z: 648.6289) and b) ceramide-1-phosphate ([C_20_H_40_NO_6_P], m/z: 506.3605). The upper panel represents the normal tissue, while the lower illustrates the wound environment at the inflammation stage
